# Comparison of efficacy of anti-diabetics on non-diabetic NAFLD: A network meta-analysis

**DOI:** 10.3389/fphar.2022.1096064

**Published:** 2023-01-09

**Authors:** Dachuan Jin, Zhongfeng Cui, Shunqin Jin, Tao Zhou, Baoqiang Guo, Peng Gao, Guangming Li

**Affiliations:** ^1^ Department of Liver Disease, Henan Provincial Infectious Disease Hospital, Zhengzhou, China; ^2^ Clinical Lab, Henan Provincial Infectious Disease Hospital, Zhengzhou, China; ^3^ Department of Radiology, Hebei Medical University, Shijiazhuang, China; ^4^ Department of Gastroenterology, Qilu Hospital of Shandong University, Jinan, China; ^5^ Department of Life Sciences, Faculty of Science and Engineering, Manchester Metropolitan University, Manchester, United Kingdom

**Keywords:** hypoglycemic agents, NAFLD, randomized controlled trials, network meta-analysis, efficacy, comparison

## Abstract

**Objective:** This study aimed to assess the efficacy of currently used anti-diabetic medications in the treatment of non-alcoholic fatty liver disease (NAFLD) without diabetes. DESIGN: The efficacy of various anti-diabetic medicines on non-alcoholic fatty liver disease in the absence of diabetes was evaluated by searching Pubmed, Embase, Cochrane Library, and Web of Science for randomized controlled trials (RCT) only. The methodological quality was evaluated using the Revised Cochrane risk-of-bias tool for randomized trials (RoB2), and the data were analyzed using Stata software (version 15.1). Results: All papers published between the time of the pooling and September 2022 were searched. There were a total of 18 randomized controlled studies with a total sample size of 1141 cases. The outcomes of interest included variations in alanine transaminase (ALT) and aspartate transaminase (AST). Rosiglitazone (SUCRA: 100%) and vildagliptin (SUCRA: 99.9%) were the best anti-diabetic medicines to improve ALT and AST, respectively, in patients with NAFLD without diabetes, according to the findings of this network meta-analysis. Conclusion: In accordance with the Network Ranking plot, Rosiglitazone was the best anti-diabetic medicine for improving ALT, and vildagliptin was the best for improving AST in patients with non-diabetic NAFLD.

## 1 Introduction

Non-alcoholic fatty liver disease (NAFLD) refers to the disease in which the liver fat content exceeds 5%, and excludes the secondary causes of alcohol, infection, drugs or other specific metabolic diseases (Zou et al., 2018). It is a chronic liver disease related to obesity, type 2 diabetes, hyperlipidemia and other diseases. 30 years ago NAFLD was an uncommon liver illness, however, it is presently the most common chronic liver disease globally, especially in western countries, the Middle East and South American countries, with an incidence rate as high as 20%–40% (Younossi et al., 2016; Younossi et al., 2019; Ge et al., 2020; Paik et al., 2020; Younossi et al., 2020; Okamura et al., 2021). As estimated, about 25% of the world’s adults have non-alcoholic fatty liver disease (Younossi et al., 2016; Younossi et al., 2019). The analysis also found that the incidence of NAFLD has been rising steadily worldwide in the 30 years from 1991 to 2020, especially among young people aged 18–39 (Zhou et al., 2019; Cotter and Rinella, 2020; Ge et al., 2020; Younossi et al., 2020; Ito et al., 2021; Henry et al., 2022). Even in countries like Japan where people live long, the incidence rate of NAFLD is increasing. It is expected that by 2040, the incidence rate of NAFLD in Japan may reach 45% (Ito et al., 2021). In addition, cirrhosis occurs in .25%–3.2% of patients with NAFLD each year, while hepatocellular carcinoma occurs in .3%–2.6% of patients with cirrhosis (D'Avola et al., 2016). Currently, NAFLD has become one of the greatest health threats of the 21st century and its treatment is rapidly becoming a worldwide concern (Lazarus et al., 2020; Ding et al., 2022).

Unfortunately, there is still no international consensus on the pharmacological treatment of NAFLD, and national guidelines or strategies for NAFLD are rare, for example, the United States Food and Drug Administration (FDA) has not approved any specific drugs for the treatment of NAFLD in the United States (Snyder et al., 2018; Elhence and Shalimar, 2020; Mantovani and Dalbeni, 2021; Petroni et al., 2021). However, the clinical treatment options include anti-diabetics, antioxidants (e.g., vitamin E, etc.) and hypolipidemics (Lazarus et al., 2020; Mantovani and Dalbeni, 2021; Rowe et al., 2022), etc. Anti-diabetic drugs have been increasingly tried as one of the commonly used pharmaceutical treatments besides the adjustment of diet and living habits for non-diabetic NAFLD patients in recent years, because it is widely accepted that insulin resistance may play an important role in the pathogenesis of NAFLD and there is a bidirectional interaction between NAFLD and type 2 diabetes NAFLD (Mazza et al., 2012; Lonardo et al., 2019; Pennisi et al., 2019; Liu et al., 2020; Sumida et al., 2020). Clinically applied anti-diabetic drugs, other than insulin, can be divided into 7 categories, including: biguanides, sulfonylureas, thiazolidinediones (TZDs) [i.e. Peroxisome proliferator activated receptor γ(PPAR-γ) agonists], glucagon-like peptide 1 receptor agonists (GLP-1RA), α-glycosidase inhibitors, dipeptidyl peptidase-4 inhibitors (DPP-4i), sodium-glucose cotransporter-2 inhibitor (SGLT2i). Transaminases, to some extent, reflect the activity of liver inflammation in patients with NAFLD and remain the most recognized classical index for evaluating liver inflammatory activity and hepatocyte destruction form various causes (Sanal, 2015). Just like alanine transaminase (ALT) and aspartate transaminase (AST) were chose as outcomes of interest in Ng’s network meta-analysis study on NAFLD, the same index were chosen in our study on non-diabetic NAFLD (Ng et al., 2022). According to previous studies, several anti-diabetic drugs have been found to improve liver function in NAFLD patients without diabetes and to improve hepatic steatosis, hepatocyte ballooning, and inflammatory activity. Because there is no consensus international guidance, and few people have made a comprehensive comparison of the efficacy of different hypoglycemic drugs for non-alcoholic fatty liver, it is not conducive to the choice of drugs in clinical work. This is why we apply this network meta-analysis.

## 2 Materials and methods

The Preferred Reporting Items for Systematic Reviews and Meta-Analyses for Network Meta-Analysis (PRISMA-NMA) statement was followed when doing this network meta-analysis, and the protocol was registered and posted on the INPLASY website at https://inplasy.com/inplasy-2022-11-0014/(INPLASY registration number: INPLASY2022110014).

### 2.1 Search strategy

Four electronic database (Pubmed, Embase, Cochrane Central Register of Controlled Trials, and Web of Science) were searched in this study from their inception through September 2022. The PICOS tool served as the foundation for the search strategy: (P) Population: non-diabetic people with NAFLD; (I) Intervention: anti-diabetic drugs; (C) Comparator: control group with placebo or usual care only; (O) Outcomes: serum biochemical tests of ALT and AST levels; (S) Study type: randomized controlled trials. Table 1 provides an outline of the comprehensive search strategy (Pubmed is used as an example).

**TABLE 1:** Search strategy on PubMed.

#1	“Non-alcoholic fatty liver disease” (MeSH))
#2	“Non-alcoholic fatty liver disease” (Title/Abstract) OR “NAFLD” (Title/Abstract) OR “non-alcoholic fatty liver disease” (Title/Abstract) OR “fatty liver non-alcoholic” (Title/Abstract) OR ((“fatty liver” (MeSH Terms) OR (“Fatty" (All Fields) AND “Liver” (All Fields)) OR “fatty liver” (All Fields)
#3	#1 OR #2
#4	“Hypoglycemic agents” (MeSH)
#5	“Hypoglycemic agents" (MeSH Major Topic) OR “agents hypoglycemic” (Title/Abstract) OR “hypoglycemic agent” (Title/Abstract) OR “agent hypoglycemic” (Title/Abstract) OR “antihyperglycemic agent” (Title/Abstract) OR “agent antihyperglycemic” (Title/Abstract) OR “Antihyperglycemics” (Title/Abstract) OR “Hypoglycemic” (Title/Abstract)
#6	#4 OR #5
#7	Randomzied controlled trials (Publication Type)
#8	#3 AND #6 AND #7

### 2.2 Inclusion criteria

Studies were included if they met all of the following criteria for inclusion: (Zou et al., 2018): Patients who have been diagnosed with NAFLD meeting the guidance from the American Association for the Study of Liver Diseases (Chalasani et al., 2018); (Okamura et al., 2021) Drug treatment using any anti-diabetic medications. Younossi et al., (2016) Clinical randomized controlled study with a control group that only received usual care in lifestyle or a placebo; (Younossi et al., 2019) Clearly reported outcome indicators that included at least one of the following: Serum ALT and AST.

### 2.3 Exclusion criteria

Studies that fit one or more of the following descriptions will be disqualified: (Zou et al., 2018): Animal models; (Okamura et al., 2021) Studies that use unreported or incomplete data; (Younossi et al., 2016) Non-randomized controlled trials; (Younossi et al., 2019) Articles that are duplicates; (Paik et al., 2020) Conference abstracts, cross-sectional studies, retrospective studies, Reviews; (Younossi et al., 2020) Patients with fatty liver brought on by alcohol or other recognized factors; (Ge et al., 2020) Patients with diabetes were included in the studies. Ito et al., (2021) Patients must be at least 18 years old.

### 2.4 Study selection

Using the literature management tool Endnote, the literature was vetted and excluded. First, two investigators independently looked for duplicates, non-randomized controlled trial studies, conference papers, review protocols, papers, and communications in the titles of the literature. Then the literature abstracts were evaluated to determine what should be included in the study and what should be excluded. The remaining papers in their entirety were examined before further studies were selected for inclusion. All literature during this process was separately reviewed, and then a comparison was made to see whether they were the same or not. Any disagreements were resolved by group discussion.

### 2.5 Data extraction

Using a seven-item, standardized, and pre-selected data extraction form, all data were collected for inclusion in the study under the following headings: (Zou et al., 2018): author, (Okamura et al., 2021), year of publication, (Younossi et al., 2016), country, (Younossi et al., 2019), study period, (Paik et al., 2020), sample size, (Younossi et al., 2020), mean age, (Ge et al., 2020), intervention, and (Ito et al., 2021) reported endpoints of interest.

### 2.6 Outcome measurement

The essential result of interest in our review was changes in mean ALT level, and secondary result of interest for our review was mean changes in AST.

### 2.7 Risk of bias of individual studies

TZ and BG autonomously evaluated the risk of bias using Revised Cochrane risk-of-bias tool for randomized trials (RoB2) for assessing the risk of bias. This tool evaluates the risk of bias with five domains considered: (Zou et al., 2018): randomized process; (Okamura et al., 2021) deviations from intended interventions; (Younossi et al., 2016) missing outcome data; (Younossi et al., 2019) measurement of the outcome; (Paik et al., 2020) selection of the reported result. Overall bias was defined as “low risk of bias” if all domains were rated as low risk, “some concerns” if at least one domain was rated as having some concerns, and “high risk of bias” if one or more domains rated as high risk or multiple domains were rated as having some concerns that might affect the validity of the results.

### 2.8 Data analysis

All variables are continuous and given as means with standard deviation (SD) in studies where anti-diabetic medications constitute the intervention (Huang et al., 2021; Theodoridis et al., 2022). The study will report continuous variables as mean difference (MD = absolute difference between the means of two groups, defined as the difference in means between the treatment and control groups and calculated with the same scale) or standardized mean difference (SMD = mean difference in outcome between groups/standard deviation of outcome between subjects, used to combine data when trials with different scales), with 95% confidence intervals (CI) and analysis. We adopted a random effects model for analysis as opposed to a fixed effects model since there are undoubtedly potential variances between studies (Jackson et al., 2011).

According to the PRISMA NMA instruction manual, we utilized Stata software (version 15.1) to aggregate and analyze NMA data using Markov chain Monte Carlo simulation chains in a Bayesian framework (Moher et al., 2015; Vats et al., 2019). Consistency was determined using Stata software, if the *p*-value is more than .05, the consistency test is said to be passed (Salanti et al., 2011). To display and describe network diagrams of various interventions, Stata software was utilized. The lines connecting the nodes in the resulting network diagrams reflect direct head-to-head comparisons between interventions, and each node represents a separate anti-diabetics intervention and a different control condition. The width of the connecting lines and the size of each node are proportional to the number of studies (Chaimani et al., 2013).

AP score was used to summarize and describe the intervention hierarchy. The P score, which averages across all competing treatments, is regarded as a frequentist analogue to surface under the cumulative ranking curve (SUCRA) values and quantifies the degree of certainty that one treatment is superior to another. The P score has a range of 0–1, with 0 denoting the worst treatment and 1 denoting the best therapy with no uncertainty. Although the P score or SUCRA can be advantageously translated into the percentage of effectiveness or acceptability of the interventions, such ratings should be regarded cautiously unless there are real clinically significant differences between interventions (Marotta et al., 2020). A network funnel plot was created and visually examined using the symmetry criterion to assess for the presence of bias resulting from small-scale investigations, which may result in publication bias in NMA (Khera et al., 2016). The potential impact of publication bias on the study’s findings was also investigated using Egger’s test and Begg’s test.

## 3 Results

### 3.1 Study and identification and selection

**FIGURE 1:**
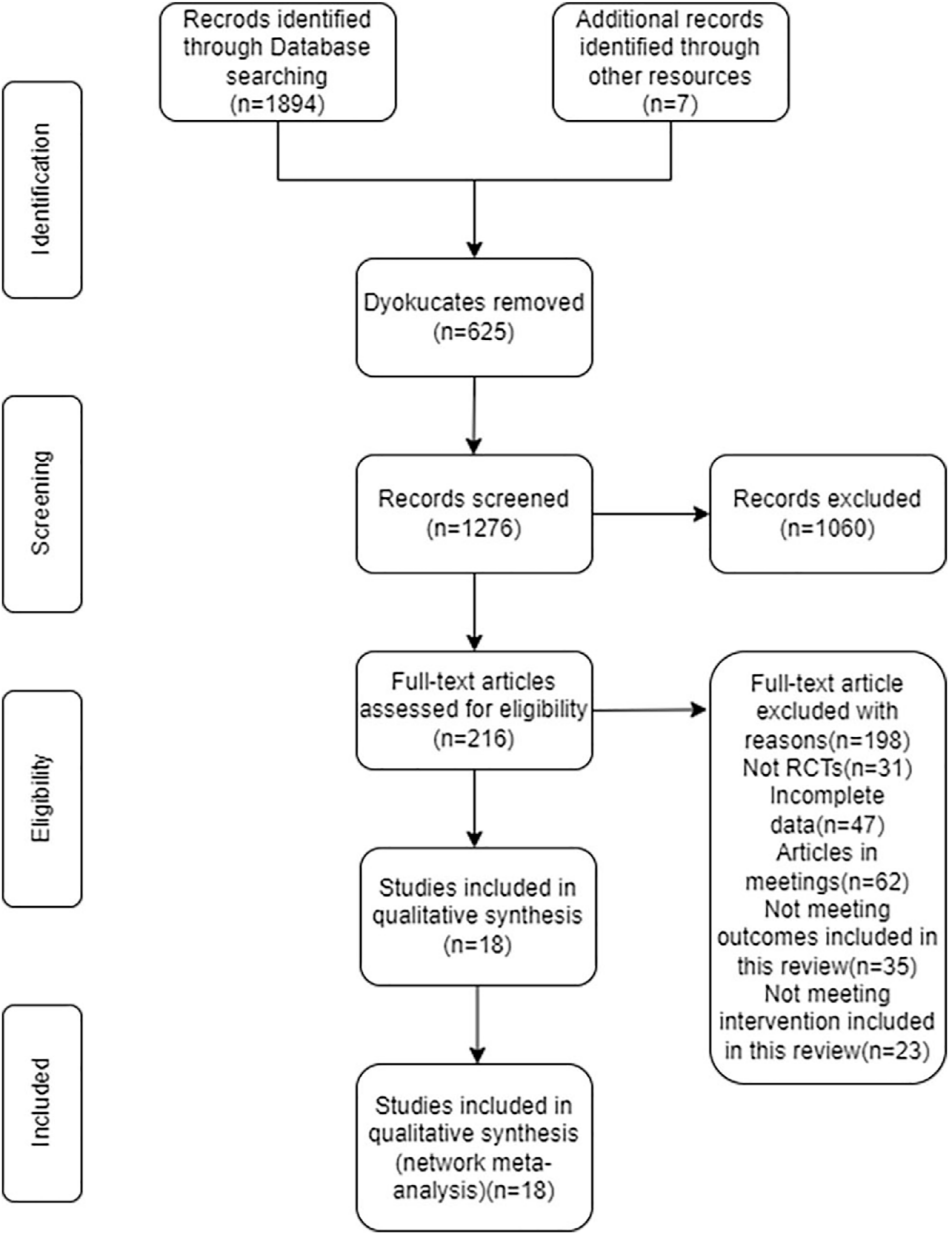
Flow diagram of literature selection. This figure showed the procedure for including RCTs with participants who have non-diabetic NAFLD.

The search of the computerized database turned up a total of 1,901 papers, while the manual search turned up nine additional things. After removing the duplicates, the remaining 1,276 papers were reviewed, and by reading the titles and abstracts of those documents, another 1,060 documents were eliminated from consideration. After carefully reading the full texts of the remaining 216 papers, 198 of them were disqualified once more (for reasons such as non-randomized controlled trials, insufficient data, conference papers, and failure to meet the interventions covered in this review), which resulted in only 18 papers being considered for this research. (Figure 1).

### 3.2 Quality assessment of the included studies

According to the Revised Cochrane risk-of-bias tool for randomized trials, the majority of the studies were rated as having a low or unclear risk of bias across the five domains. Five studies was determined to have a high risk of bias due to deviations from intended interventions (Uygun et al., 2004; Jin et al., 2010; Yaghoubi et al., 2017; Taheri et al., 2020; Doustmohammadian et al., 2022). Measurement of the outcome was deemed to be a high risk of bias in three studies (Cui et al., 2006; Yaghoubi et al., 2017; Khoo et al., 2019). Two studies was found to have a high risk of bias due to selection of the reported result (Rana et al., 2016; Lee et al., 2022). Supplementary Figures S1 and Supplementary Figure S2 show the risk-of-bias assessment of the trials included in this study.

### 3.3 Characteristics of the included studies

The papers from 18 randomized controlled trials, totaling 1,141 individuals with non-diabetic NAFLD, were included in our analysis. They were all published between 2004 and 2022. Empagliflozin (2 studies) (Taheri et al., 2020; Lee et al., 2022), Liraglutide (1 studies) (Khoo et al., 2019), Metformin (9 studies) (Uygun et al., 2004; Garinis et al., 2010; Hajiaghamohammadi et al., 2012; Sanchez-Munoz et al., 2013; Soifer et al., 2015; Rana et al., 2016; Shahebrahimi et al., 2017; Anushiravani et al., 2019; Mohammadi et al., 2022), Pioglitazone (7 studies) (Aithal et al., 2008; Jin et al., 2010; Hajiaghamohammadi et al., 2012; Rana et al., 2016; Shahebrahimi et al., 2017; Yaghoubi et al., 2017; Anushiravani et al., 2019), Rosiglitzone (1 study) (Cui et al., 2006), Sitagliptin (1 study) (Doustmohammadian et al., 2022), and Vildagliptin (1 study) (Hussain et al., 2016) were interventions involved in our analysis. Eighteen research reported using ALT as an outcome indicator while fifteen studies used AST. There were two studies from East Asia (Cui et al., 2006; Jin et al., 2010), nine from West Asia (Uygun et al., 2004; Hajiaghamohammadi et al., 2012; Soifer et al., 2015; Shahebrahimi et al., 2017; Yaghoubi et al., 2017; Anushiravani et al., 2019; Taheri et al., 2020; Doustmohammadian et al., 2022; Mohammadi et al., 2022), two from South Asia (Hussain et al., 2016; Rana et al., 2016), one from Southeast Asia (Khoo et al., 2019), one from North America (Lee et al., 2022), one from South America (Sanchez-Munoz et al., 2013), one from South Europe (Garinis et al., 2010), and one from West Europe (Aithal et al., 2008). Supplementary Table S1 displays the characteristics of the included studies.

### 3.4 Network meta-analysis

**FIGURE 2:**
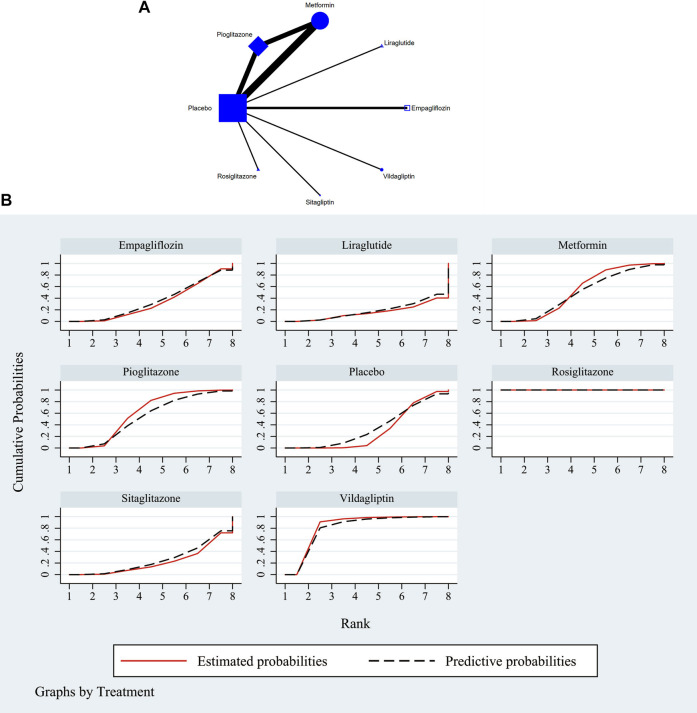
**(A)** NMA figure for ALT. Each node stands for a different treatment. The size of each node is based on how many people were given that treatment. The lines show direct comparisons, and the width of the line depends on how many trials were done. **(B)** SUCRA plot for ALT. The cumulative rank likelihood of each therapy is represented by the area under the curve, with bigger areas denoting higher probabilities.

**FIGURE 3:**
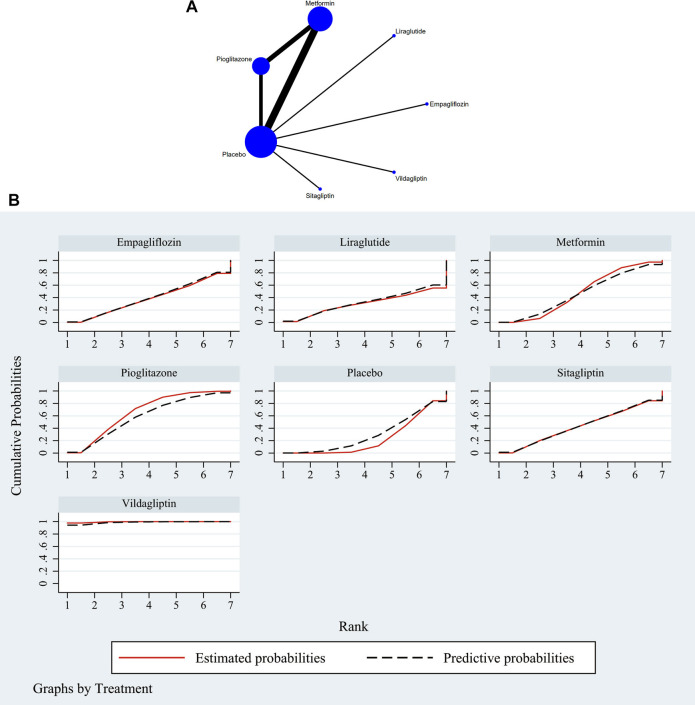
**(A)** NMA figure for AST. Each node stands for a different treatment. The size of each node is based on how many people were given that treatment. The lines show direct comparisons, and the width of the line depends on how many trials were done. **(B)** SUCRA plot for AST. The cumulative rank likelihood of each therapy is represented by the area under the curve, with bigger areas denoting higher probabilities.

The full NMA figure were shown in Figure 2A and Figure 3A. The nodes represent comparative therapy, while the lines indicate which therapies were compared. The number of participants in each node determined the size of the nodes. The connecting line’s thickness was proportional to the number of trials in each comparison.

#### 3.4.1 Improvement of ALT in NAFLD

The consistency and inconsistency of all *p*-values for indirect and direct comparisons between all studies were assessed, and nearly all *p*-values were greater than .05, indicating that the effect of study consistency was acceptable. Information on consistency and inconsistency tests is displayed in Supplementary Table S2 and Supplementary Table S4.

According to the Network meta-findings, analysis’s rosiglitazone [MD = -307.80, 95% CI =(−372.15, −243.45)], vildagliptin [MD = −23.40, 95% CI =(−41.65, −5.15)], pioglitazone [MD = −7.03, 95% CI =(−15.15, 1.08)], metformin [MD = −5.23, 95% CI =(−12.45, 2.00)], and empagliflozin [MD = .26, 95% CI =(−-12.53, 13.05)] all outperformed the control group in lowering serum ALT levels when compared to the routine measures used by the control group. Sitagliptin [MD = 5.00, 95% CI = (−12.60, 13.11)] and liraglutide [MD = 5.00, CL= (−12.62, 22.62)] did not do as well in lowering serum ALT levels as the control group did. According to the SUCRA, the likelihood rating of the various interventions in terms of lowering ALT level, Rosiglitazone was given priority (SUCRA: 100% as indicated in Figure 2B). The comparison of the two distinct therapies will be presented in Table 2 and Supplementary Table S6.

**TABLE 2:** League table on ALT.

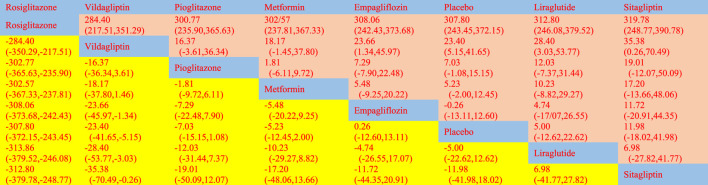

#### 3.4.2 Improvement of AST in NAFLD

The consistency and inconsistency of all *p*-values for indirect and direct comparisons between all studies were assessed, and nearly all *p*-values were greater than .05, indicating that the effect of study consistency was acceptable. Information is displayed in Supplementary Table S3 and Supplementary Table S5.

The network meta-analysis revealed that vildagliptin [MD = −19.70, 95% CI=(−29.79, −9.61)], pioglitazone [MD = −4.51, 95%CL=(−9.19, −.16)], sitagliptin [MD = −2.00, 95% CI=(− 10.72, 6.72)], metformin [MD = −2.61, 95% CI=(−6.41, 1.39)], empagliflozin [MD = −1.20. 95% CI =(−10.42, 8.01)], and liraglutide [MD = 1.00, 95% CI=(−14.74, 16.74)] all outperformed the placebo group in lowering serum AST levels in non-diabetics when compared to the control group for routine measures. Vildagliptin was placed top in the SUCRA for the likelihood ranking of the various anti-diabetic medications in terms of lowering blood AST concentration (SUCRA: 99.9% as shown in Figure 3B). The comparison of the two distinct therapies will be presented in Table 3 and Supplementary Table S7.

**TABLE 3:** League table on AST.

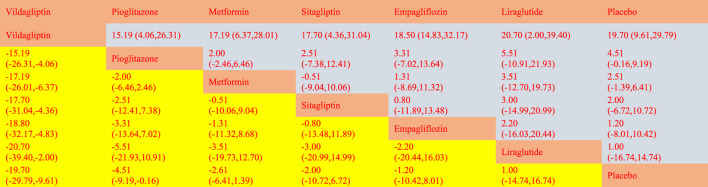

### 3.5 Publication bias test

**FIGURE 4:**
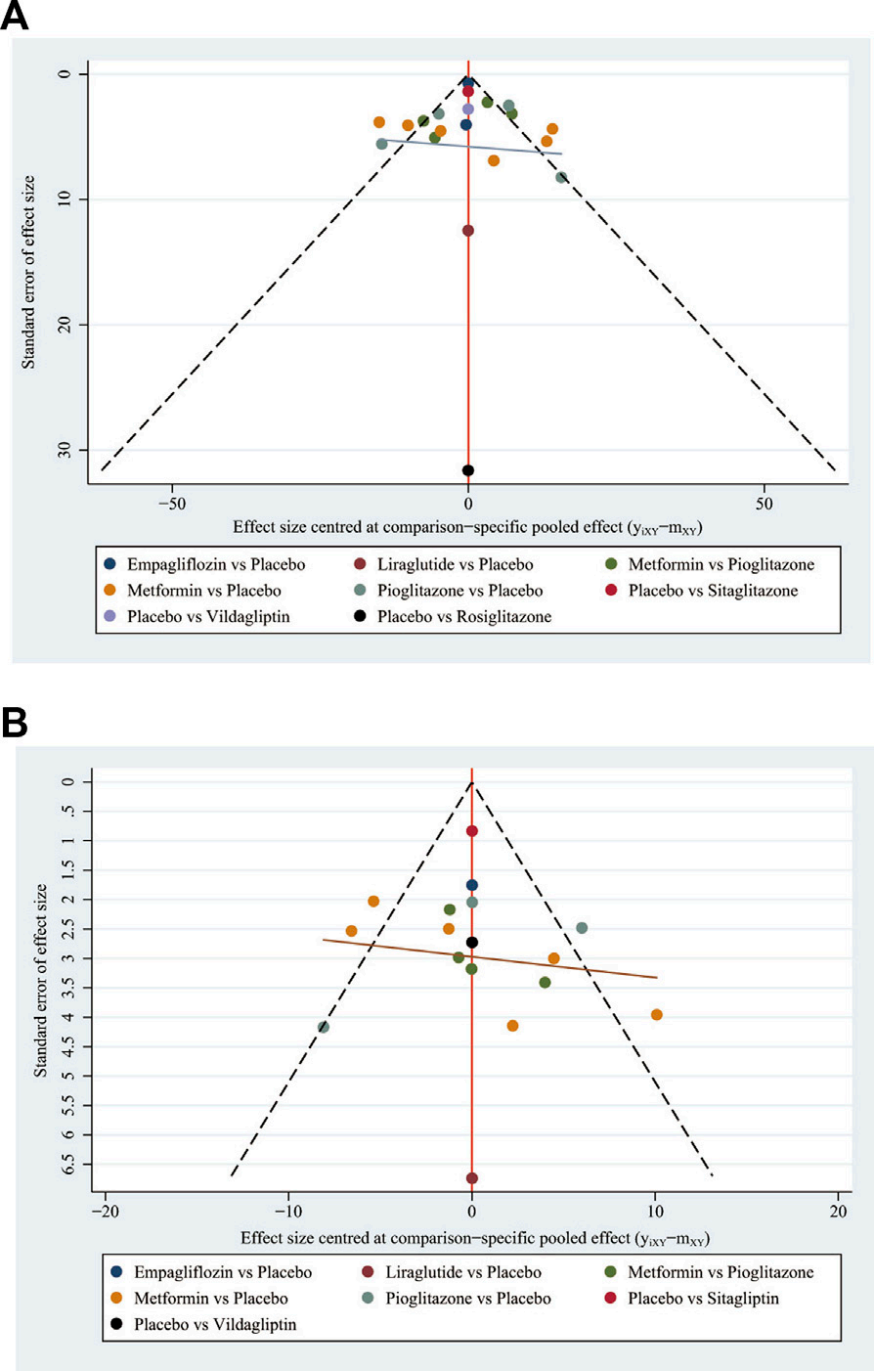
Funnel plot on publication bias **(A)** ALT. An asymmetrical graph suggested that there might be publishing bias while a symmetrical graph showed no clear publication bias. Funnel plot on publication bias **(B)** AST. An asymmetrical graph suggested that there might be publishing bias while a symmetrical graph showed no clear publication bias.

To investigate any potential publication bias, we created separate funnel plots for each outcome variable. Funnel plots did not show any notable publication bias (Wallace et al., 2009). Specifics as displayed in Figures 4A,B. In addition, the *p*-values from Egger’s and Begg’s test for ALT were .604 and .409, while the *p*-values for Egger’s test and Begg’s test of AST were .805 and .636 respectively. No indication of publication bias was bound.

## 4 Discussion

In this study, we compared the efficacy of seven currently available clinically applied anti-diabetic treatments by combining literature searches to find 18 studies which met the inclusion criteria. Consistent with most previous similar studies, the outcomes of interest that we compared are improvements in two biochemical markers, including ALT and AST, in patients with non-diabetic NAFLD (Theodoridis et al., 2022). Studies on ALT and AST, with 1,141 and 908 patients enrolled, respectively, represent a large sample size. Our findings suggest that Rosiglitazone is the best anti-diabetic drug for improving ALT, while Vildagliptin is the best drug for improving AST. It is well known that ALT has long been the most classic, commonly used and surrogate indicator of hepatocyte damage (Kallai et al., 1964; Calvaruso and Craxì, 2009; Sanal, 2015). ALT is mainly distributed in the cytoplasm of liver cells, while AST is distributed in the mitochondria of hepatocytes as a mitochondrial enzyme, but it is also present in cardiac muscle, skeletal muscle, kidney, brain, pancreas, lung, leukocytes, however, with much lower activity.

The studies that met the inclusion criteria for this study involved 7 drugs of 5 categories, namely biguanides (metformin), DPP-4i (vildagliptin, sitagliptin), TZD class (rosiglitazone, pioglitazone), SGLT2i (empagliflozin), and GLP-1RA (liraglutide) drugs (Ranjbar et al., 2019; Liu et al., 2020). As mentioned earlier, vildagliptin was the best AST-lowering drug and the second-ranked excellent ALT-lowering drug in our study. As a DPP-4i class drug, vildagliptin significantly reduced not only ALT levels but also intrahepatic triglyceride (TG) levels, an effect that has been confirmed by magnetic resonance examination. The specific mechanism may be through the influence of hepatic lipid metabolism and triglyceride transport (Macauley et al., 2015). The insulin resistance that is a major contributor to NAFLD is also reduced by vildagliptin. Additionally, increased expression of dipeptidyl peptidase-4 is linked to hepatic steatosis; as a DPP-4 inhibitor, vildagliptin is effective at improving NAFLD (Hussain et al., 2016). In combination with our findings, it follows then that vildagliptin might be the best option for people who have abnormal liver transaminases, notably high AST (Macauley et al., 2015).

Another drug with excellent efficacy is pioglitazone, which is the only anti-diabetic drug recommended by a clinical guideline for the treatment of NASH (Chalasani et al., 2018; Kawaguchi-Suzuki et al., 2018; Mantovani and Dalbeni, 2021). In our study, pioglitazone was second only to vildagliptin in improving AST and second only to rosiglitazone and vildagliptin in improving ALT. Both pioglitazone and rosiglitazone belong to TZD functioning as highly selective PPAR-γ agonists, which is a key factor in the regulation of glucose and lipid metabolism (Francque et al., 2021). Activation of PPAR-γ increases adipocyte uptake of free fatty acids, protecting the liver, skeletal muscle, and beta cells against the deleterious metabolic effects of lipid poisoning (Mookkan et al., 2014). PPAR-γ receptors are widely distributed in adipose tissue and hepatic Kupffer cells, which are associated with liver fibrosis (Raschi et al., 2018; American Diabetes Association, 2019; Francque et al., 2021). Pioglitazone can regulate the production and release of different adipokines, including adiponectin, tumor necrosis factor-α, and monocyte chemoattractant protein-1. Its effect on NALFD may be a result of its immunomodulatory and anti-inflammatory properties. Previous studies have found that pioglitazone results in significant histological improvement of inflammation and steatosis in NASH with or without diabetes, but there is controversy about whether it improves liver fibrosis (Cusi et al., 2016; Pennisi et al., 2019; Kumar et al., 2021).

As the best drug to improve ALT in the study, rosiglitazone of the thiazolidinedione class drugs is the optimal choice among anti-diabetic drugs. The same as pioglitazone, this mechanism of action may be because thiazolidinedione drugs can improve the synthesis and uptake of fatty acids in adipose tissue, and transfer the load of free fatty acids load from liver to adipocytes (Raza et al., 2021). This improves fat accumulation in liver and improves liver function. In Hockings and Tahan’s studies, in addition to ameliorating liver inflammation and insulin resistance in methionine- and choline-deficient diet-induced steatosis in Wistar rats, rosiglitazone has been shown to reverse hepatic steatosis and reduce intramyocellular lipids in Zucker fatty rats (Hockings et al., 2003; Tahan et al., 2007). Our finding is consistent with previous studies proposing that thiazolidinediones drugs are the most powerful drugs for the treatment of non-alcoholic steatohepatitis (Vuppalanchi and Chalasani, 2009; Raza et al., 2021).

Interestingly, Mookkan discovered that hepatic steatosis and TGs were significantly reduced in the mice treated with vildagliptin and rosiglitazone combo (Mookkan et al., 2014). However, clinical investigations are necessary to establish this finding. In addition, because PPAR-γ is also present and expressed in blood vessels, Rosiglitazone has been reported to have a regulatory effect on vascular homeostasis in animal studies and to have a protective effect on patients with arteriosclerosis, which can prevent the accumulation of macrophages in damaged arteries and reduces the expression of inflammatory factors such as tumor necrosis factor. But further clinical trials are needed to confirm this effect of rosiglitazone (Han et al., 2017). Since NAFLD is closely associated with cardiovascular diseases such as coronary heart disease and atherosclerosis, this effect of rosiglitazone is of clinical importance (Polyzos et al., 2021).

Overall, our study does have some bearing on clinical practice. We confirmed that Rosiglitazone of TZD class and vildagliptin of DPP-4i class are preferred for improving ALT and AST, respectively, for the treatment of NAFLD in patients who do not have diabetes. This finding is in line with the findings of previous studies that looked at those two medications on their own. However, it is particularly important to note here that even the same category of drugs may have relatively large differences in efficacy. For example, the efficacy of sitagliptin, which also belongs to DPP-4i drugs, differs significantly from that of vildagliptin in our study. Vildagliptin has a much better efficacy in improving transaminase, especially ALT.

## 5 Strengths and limitations

First, a relatively high sample size of 1,141 patients from 18 studies was included in our analysis. We performed the first network meta-analysis evaluating the efficacy of anti-diabetic medications in patients with non-diabetic NAFLD. Through direct and indirect comparative evidence analysis, the investigation comprised seven medications from five major classes and gave more complete recommendations for clinical practice application by medical experts.

Second, the findings of our investigation are not without their flaws. When we include the studies’ original data, we make every effort to control the heterogeneity of the research; yet, it was unavoidable for there to be variability between the studies (for example, patients came from different countries, regions, races in the world, and studies with different gender ratio).

Third, in our research, it is important for readers to use caution when interpreting these data. This is due to the fact that there are not yet a sufficient number of research that are focused on particular therapies. The anti-diabetic medications that are currently being used in clinical practice primarily contain more than 30 pharmaceuticals that fall into 7 different groups. However, all of the included studies only covered 7 drugs from 5 major classes, limited by current research progress.

Finally, the indicators of the study only include ALT and AST, because we found it difficult to accurately quantify the improvement of non-diabetic NAFLD after treatment with anti-diabetics through uniform imaging and/or histological examination. This led to the fact that the data of some studies with different evaluation methods cannot be included and compared effectively. We would focus on finding a better quantitative evaluation of the therapeutic effect of NAFLD, which can detect even if there is a slight improvement only. In addition, more large-scale and comprehensive clinical studies of anti-diabetic drugs are needed in the future to obtain sufficient evidence for more direct and comprehensive comparisons of drug efficacy in the treatment of patients with NAFLD without diabetes.

## 6 Conclusion

Based on the results of our study, we suggest that patients with NAFLD without diabetes mellitus can be treated with rosiglitazone or vildagliptin in reference to the patient’s laboratory test results when choosing a drug therapy to improve ALT and/or AST levels.

## Data Availability

The original contributions presented in the study are included in the article/Supplementary Material, further inquiries can be directed to the corresponding authors.
