# Supplementary material for: Comparison of efficacy of anti-diabetics on non-diabetic NAFLD: A network meta-analysis
Source: Front Pharmacol. 2023 Jan 9;13:1096064. doi: 10.3389/fphar.2022.1096064 (PMC9868463; doi:10.3389/fphar.2022.1096064)
Supplement: Supplementary file 1 [file DataSheet1.pdf]

Supplementary Material

1 Supplementary Figures

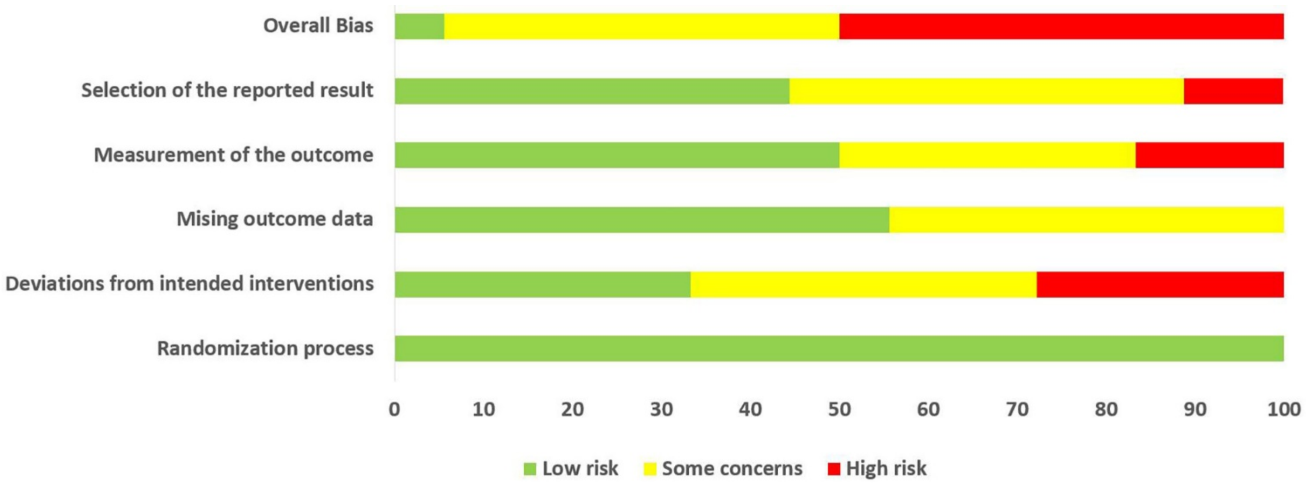

Figure S1. Risk of bias graph assessed with RoB2: review authors' judgement about each risk of bias item presented as percentages across all included studies in this trial

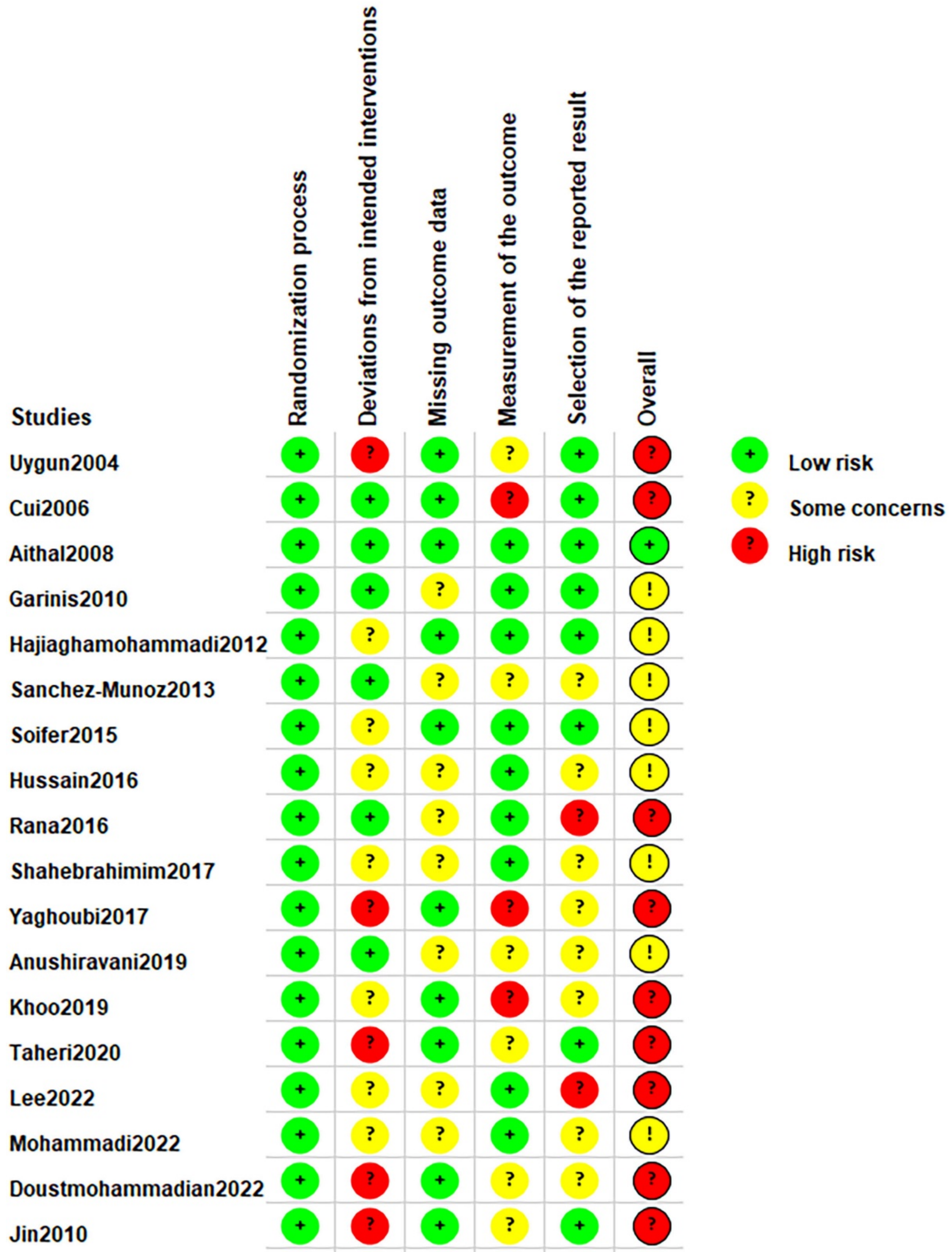

Figure S2. Risk of bias summary assessed with RoB2: review authors' judgements about each risk of bias item for each included study in this trial

2. Supplementary Tables

Table S1. Characteristics of the Studies included in the Network Meta-analysis

Author (Year/Country)	Age （mean+SD	Entry No.	Final No.	Intervention		Outcome of interest
				Experiment	Control	
Doustmohammadian(1) (2022/Iran)	T:45.0(4.1)<br>C:39.0(2.6)	T:60<br>C:60	T:44<br>C:32	Sitagliptin<br>Dosage:30mg<br>Freq.:1time/d<br>Duration:56wks	Placebo	ALT,AST
Mohammadi(2) (2022/Iran)	T:45.03(12.4 4)<br>C:44.4(2.07)	T:37<br>C:35	T: 35<br>C: 35	Metformin<br>Dosage:500mg<br>Freq.:1time/d<br>Duration:6mons	Placebo	ALT,AST
Lee(3) (2022/USA)	T: 50.4(2.3)<br>C: 53.1(2.2)	T: 18<br>C: 17	T: 18<br>C: 17	Empagliflozin<br>Dosage:10mg<br>Freq.:1time/d<br>Duration:3mons	Placebo	ALT
Taheri(4) (2020/Iran)	T: 43.8(9.7)<br>C: 44.1(9.3)	T: 50<br>C: 50	T: 43<br>C: 47	Empagliflozin<br>Dosage:10mg<br>Freq..:1time/d<br>Duration:24wks	Placebo	ALT.AST
Khoo(5) (2019/Singapore)	T: 38.6(8.2)<br>C: 43.6(9.9)	T: 15<br>C: 15	T: 14<br>C: 13	Liraglutide<br>Dosage:3mg<br>Freq.:1time/d<br>Duration:26wks	DE	ALT,AST
Anushiravani(6) (2019/Iran)	T1: NA<br>T2: NA<br>C: NA	T1: 30<br>T2: 30<br>C: 30	T1: 30<br>T2: 30<br>C: 30	Lifestyle + Pioglitazone<br>Dosage:15mg<br>Freq.:1time/d<br>Duration:3mons<br><br>Lifestyle+ Metformin<br>Dosage:500mg<br>Freq.:1time/d<br>Duration:3mons	Lifestyle+Placebo	ALT,AST
Yaghoubi(7) (2017/Iran)	T: 35(7)<br>C: 38(10)	T: 30<br>C: 30	T: 30<br>C: 30	Pioglitazone<br>Dosage:30mg<br>Freq.:1time/d<br>Duration:12wks	Placebo	ALT,AST
Shahebrahimim(8) (2017/Iran)	T: NA<br>C: NA	T: 31<br>C: 31	T: 31<br>C: 31	Metformin<br>Dosage:2000mg<br>Freq.:1time/d<br>Duration:12wks	Pioglitazone<br>Dosage:30mg<br>Freq.:1time/day<br>Duratin:12wks	ALT,AST
Rana(9) (2016/India)	T: NA<br>C: NA	T: 31<br>C: 33	T: 31<br>C: 33	Metformin<br>Dosage: N/A<br>Freq.: N/A<br>Duration: N/A	Pioglitazone<br>Dosage: N/A<br>Freq.: N/A<br>Duration: N/A	ALT,AST

Hussain(10)<br>(2016/Pakistan)	T: 28(15)<br>C: 31(12)	T: 29<br>C: 29	T: 29<br>C: 29	Vildagliptin<br>Dosage:50mg<br>Freq.:1time/d<br>Duration:4mons	Placebo	ALT,AST
Soifer(11)<br>(2015/Israel)	T: 51.9(10.9)<br>C: 55.2(14.0)	T: 32<br>C: 31	T: 27<br>C: 25	Metformin<br>Dosage:850-1700mg<br>Freq.:1time/d<br>Duration:4mons	Placebo	ALT,AST
Sanchez-Munoz(12)<br>(2013/Mexico)	T: 39.50(6.0)<br>C: 38.5(8.5)	T: 9<br>C: 10	T: 8<br>C: 8	Metformin<br>Dosage:1000mg<br>Freq.:1time/d<br>Duration:12wks	Placebo	ALT,AST
Hajiaghamohammadi(13)<br>(2012/Iran)	T: 32.5(6.5)<br>C: 33.4 (6.6)	T: 22<br>C: 22	T: 22<br>C: 22	Metformin<br>Dosage:500mg<br>Freq.:1time/d<br>Duration:2mons	Pioglitazone<br>Dosage:15mg<br>Freq.:1time/d<br>Duration:2mons	ALT,AST
Jin(14)<br>(2010/China)	T: 50.3(12.8)<br>C: 53.7(10.1)	T: 60<br>C: 60	T: 60<br>C: 60	Pioglitazone<br>Dosage:30mg<br>Freq.:1time/day<br>Duration:6mons	Placebo	ALT,AST
Garinis(15)<br>(2010/Italy)	T: 40.8(13)<br>C: 45.8(16.6)	T: 25<br>C: 25	T: 25<br>C: 25	Diet+Metformin<br>Dosage:1000mg<br>Freq.:1time/d<br>Duration:6mons	Diet+Placebo	ALT,AST
Aithal(16)<br>(2008/UK)	T: 52(10.75)<br>C: 55(11.5)	T: 37<br>C: 37	T: 37<br>C: 37	Pioglitazone<br>Dosage:30mg<br>Freq.:1time/d<br>Duration:12mons	Placebo	ALT
Cui(17)<br>(2006/China)	T: 46.2(2.6)<br>C: 45.5(2.0)	T: 63<br>C: 61	T: 63<br>C: 61	Rosiglitazone<br>Dosage:4mg<br>Freq.:2times/d<br>Duration:24wks	Placebo	ALT
Uygun(18)<br>(2004/Turkey)	T: 39.8(10.6)<br>C: 41.5(9.1)	T: 17<br>C: 17	T: 17<br>C: 17	Metformin<br>Dosage:850mg<br>Freq.:2times/d<br>Duration:6mons	Placebo	ALT,AST

Note: T: experimental group, C: control group; ALT: Alanine transaminase, AST: Aspartate transaminase, N/A: not available; DE: Intervention of diet and exercise

Supplementary Table S2. Consistency test for ALT

		Coef.	Std. Err.	z	P>|z|	[95% Conf. Interval]	
_y_B	_cons	11.72144	16.64819	0.70	0.481	-20.90841	44.3513
_y_C	_cons	-5.48314	7.518401	-0.73	0.466	-20.21893	9.252655
_y_D	_cons	-7.289748	7.751363	-0.94	0.347	-22.48214	7.902644
_y_E	_cons	-.2556842	6.558593	-0.04	0.969	-13.11029	12.59892
_y_F	_cons	-308.0556	33.48223	-9.20	0.000	-373.6795	-242.4316
_y_G	_cons	4.7444417	11.12789	0.43	0.670	-17.06584	26.55467
_y_H	_cons	-23.65558	11.38661	-2.08	0.038	-45.97294	-1.338232

Note: B: Liraglutide, C: Metformin, D: Pioglitazone, E: Placebo, F: Rosiglitazone, G: Sitaglitazone, H: Vldagliptin

Supplementary Table S3. Consistency test for AST

		Coef.	Std. Err.	z	P>|z|	[95% Conf. Interval]	
_y_B	_cons	2.201543	9.303728	0.24	0.813	-16.03343	20.43651
_y_C	_cons	-1.310306	5.10455	-0.26	0.797	-11.31504	8.694429
_y_D	_cons	-3.309706	5.271829	-0.63	0.530	-13.6423	7.022888
_y_E	_cons	1.202752	4.702059	0.26	0.798	-8.013115	10.41862
_y_F	_cons	-.7973625	6.472593	-0.12	0.902	-13.48341	11.88869
_y_G	_cons	-18.49736	6.973467	-2.65	0.008	-32.16511	-4.829618

Note: B: Liraglutide, C: Metformin, D: Pioglitazone, E: Placebo, F: Sitagliptin, G: Vildagliptin

Supplementary Table S4. Inconsistency test for ALT

		Coef.	Std.Err.	z	P>|z|	[95% Conf.Interval]	
_y_B	_cons	11.70183	16.72564	0.70	0.484	-21.07981	44.48348
_y_C	des_CE	-18.15474	10.78797	-1.68	0.092	-39.29877	2.989286
	_cons	9.691688	11.77855	0.82	0.411	-13.39384	32.77722
_y_D	des_DE	-6.935734	11.28878	-0.61	0.539	-29.06134	15.18987
	des_CD	9.593643	11.20802	0.86	0.392	-12.37367	32.56095
	_cons	-.8929866	11.59127	-0.08	0.939	-23.61145	21.82548
_y_E	_cons	-.2752334	6.623881	-0.04	0.967	-13.2578	12.70734
_y_F	_cons	-308.0751	33.52088	-9.19	0.000	-373.7748	-242.3754
_y_G	_cons	4.724877	11.24366	0.42	0.674	-17.31229	26.76204
_y_H	_cons	-23.67512	11.49978	-2.06	0.040	-46.21422	-1.13597

Note: B: Liraglutide, C: Metformin, D: Pioglitazone, E: Placebo, F: Rosiglitazone, G: Sitagliptin, H: Vildagliptin

Supplementary Table S5. Inconsistency test for AST

		Coef.	Std. Err.	z	P>|z|	[95% Conf. Interval]	
_y_B	_cons	2.197834	9.922122	0.22	0.825	-17.24917	21.64484
_y_C	des_CE	-8.00444	6.205473	-1.29	0.197	-20.16694	4.158064
	_cons	4.494011	7.708769	0.58	0.560	-10.6149	19.60292
_y_D	des_DE	-.018628	6.894597	0.00	0.998	-13.53179	13.49453
	des_CD	2.229177	6.443337	0.35	0.729	-10.39953	14.85789
	_cons	-.9036758	7.56637	-0.12	0.905	-15.73349	13.92614
_y_E	_cons	1.199107	5.295168	0.23	0.821	-9.179232	11.57745
_y_F	_cons	-.8010434	7.334282	-0.11	0.913	-15.17597	13.57389
_y_G	_cons	-18.50104	7.779877	-2.28	0.017	-33.74932	-3.252765

Note: B: Liraglutide, C: Metformin, D: Pioglitazone, E: Placebo, F: Sitagliptin, G: Vildagliptin

Supplementary Table S6. Assessment of heterogeneity for direct and indirect comparison of ALT between pair-wise and network meta-analysis

Side	Direct		Indirect		Difference		P>z	tau
	Coef.	Std. Err.	Coef.	Std. Err.	Coef.	Std. Err.		
A E *	-.2820364	6.584492	3.549567	79.10769	-3.831603	79.38126	0.962	8.891227
B E *	-12	15.32018	11.49872	632.9031	-23.49872	633.3345	0.970	8.887212
C D	-3.302809	4.975291	1.78138	7.751653	-5.084189	9.216009	0.581	9.267283
C E	4.92148	4.319877	6.563086	8.531789	-1.641605	9.566199	0.864	9.392318
D E	5.593106	5.274327	10.01849	7.445479	-4.425379	9.118759	0.627	9.322768
E F *	-307.8	32.83454	-307.2884	633.5653	-.5115851	632.8387	0.999	8.887212
E G *	5	8.993188	5.511586	632.7675	-.5115861	632.8284	0.999	8.887212
E H *	-23.4	9.31142	-22.88842	632.8364	-.511585	632.8927	0.999	8.88721

∗ Warning: all the evidence about these contrasts comes from the trials which directly compare them. Note: A: Empagliflozin, B: Liraglutide, C: Metformin, D: Pioglitazone, E: Placebo, F: Rosiglitazone, G: Sitaglitazone, H: Vldagliptin

Supplementary Table S7. Assessment of heterogeneity for direct and indirect comparison of AST between pair-wise and network meta-analysis

Side	Direct		Indirect		Difference		P>z	tau
	Coef.	Std. Err.	Coef.	Std. Err.	Coef.	Std. Err.		
A E *	1.200003	4.710889	2.089326	84.53104	-.8893232	84.6622	0.992	4.371837
B E *	-1	8.031583	3.406478	632.6037	-4.406478	632.7265	0.994	4.370394
C D	-3.769784	2.648147	3.054158	4.548644	-6.823942	5.269469	0.195	4.413035
C E	3.212062	2.308589	-.8555731	5.225918	4.067635	5.739683	0.479	4.72639
D E	2.291683	3.083147	8.226549	3.976557	-5.934866	5.058624	0.241	4.46077
E F *	-2	4.448832	-4.40604	632.51	2.40604	632.5245	0.997	4.370394
E G *	-19.7	5.150427	-22.10604	632.5152	2.406037	632.5245	0.997	4.370394

∗ Warning: all the evidence about these contrasts comes from the trials which directly compare them. Note: A: Empagliflozin, B: Liraglutide, C: Metformin, D: Pioglitazone, E: Placebo, F: Sitagliptin, G: Vildagliptin

References

1. Doustmohammadian A, Nezhadisalami A, Safarnezhad Tameshke F, Motamed N, Maadi M, Farahmand M, et al. A randomized triple-blind controlled clinical trial evaluation of sitagliptin in the treatment of patients with non-alcoholic fatty liver diseases without diabetes. Frontiers in medicine. 2022;9.

2. Mohammadi AAH, Jahromi SK, Gooraji SA, Bastani A. Comparison of the Therapeutic Effects of Melatonin, Metformin and Vitamin E on Non-Alcoholic Fatty Liver Disease: A Randomized Clinical Trial. Journal of Advances in Medical and Biomedical Research. 2022;30(140):232-40.

3. Lee MH, Neeland IJ, de Albuquerque Rocha N, Hughes C, Malloy CR, Jin ES. A randomized clinical trial evaluating the effect of empagliflozin on triglycerides in obese adults: Role of visceral fat. Metabolism open. 2022;13.

4. Taheri H, Malek M, Ismail-Beigi F, Zamani F, Sohrabi M, Reza Babaei M, et al. Effect of Empagliflozin on Liver Steatosis and Fibrosis in Patients With Non-Alcoholic Fatty Liver Disease Without Diabetes: A Randomized, Double-Blind, Placebo-Controlled Trial. Adv Ther. 2020;37(11):4697-708.

5. Khoo J, Hsiang JC, Taneja R, Koo SH, Soon GH, Kam CJ, et al. Randomized trial comparing effects of weight loss by liraglutide with lifestyle modification in non-alcoholic fatty liver disease. Liver international : official journal of the International Association for the Study of the Liver. 2019;39(5):941-9.

6. Anushiravani A, Haddadi N, Pourfarmanbar M, Mohammadkarimi V. Treatment options for nonalcoholic fatty liver disease: a double-blinded randomized placebo-controlled trial. European journal of gastroenterology & hepatology. 2019;31(5):613-7.

7. Yaghoubi M, Jafari S, Sajedi B, Gohari S, Akbarieh S, Heydari AH, et al. Comparison of fenofibrate and pioglitazone effects on patients with nonalcoholic fatty liver disease. European Journal of Gastroenterology and Hepatology. 2017;29(12):1385-8.

8. Shahebrahimi K, Zulnoorian S, Almasi A, Sharifi A, Keshvarz AA, Farshchian N. A comparison of the therapeutic effects of metformin, pioglitazone and vitamin E in patients with non-alcoholic fatty liver. Journal of Babol University of Medical Sciences. 2017;19(9):32-8.

9. Rana H, Yadav SS, Reddy HD, Singhal S, Singh DK, Usman K. Comparative effect of insulin sensitizers and statin on metabolic profile and ultrasonographical score in non alcoholic fatty liver disease. Journal of clinical and diagnostic research. 2016;10(8):OC19 - OC23.

10. Hussain M, Babar MZM, Hussain MS, Akhtar L. Vildagliptin ameliorates biochemical, metabolic and fatty changes associated with non alcoholic fatty liver disease. Pakistan Journal of Medical Sciences. 2016;32(6):1396-401.

11. Soifer E, Gavish D, Shargorodsky M. Does metformin treatment influence bone formation in patients with nonalcoholic fatty liver disease? Horm Metab Res. 2015;47(8):556-9.

12. Sanchez-Munoz V, Salas-Romero R, Del Villar-Morales A, Martinez-Coria E, Pegueros-Perez A, Franco-Sanchez JG. Decrease of liver fat content by aerobic exercise of metformin therapy in overweigth or obese women(Disminución del contenido de grasa hepática mediante ejercicio aeróbico comparado con metformina en mujeres con sobrepeso u obesidad)(Spanish). Revista De Investigacion Clinica-Clinical and Translational Investigation. 2013;65(4):307-17.

13. Hajiaghamohammadi AA, Ziaee A, Oveisi S, Masroor H. Effects of metformin, pioglitazone, and silymarin treatment on non-alcoholic fatty liver disease: A randomized controlled pilot study. Hepatitis monthly. 2012;12(8).

14. Jin H, Zhou Y, Ming K. Efficacy of pioglitazone in treatment of 60 patients with nonalcoholic steatohepatitis. Pharmaceutical care and research. 2010;10(3):221 - 3.

15. Garinis GA, Fruci B, Mazza A, De Siena M, Abenavoli S, Gulletta E, et al. Metformin versus dietary treatment in nonalcoholic hepatic steatosis: a randomized study. International journal of obesity (2005). 2010;34(8):1255-64.

16. Aithal GP, Thomas JA, Kaye PV, Lawson A, Ryder SD, Spendlove I, et al. Randomized, placebo-controlled trial of pioglitazone in nondiabetic subjects with nonalcoholic steatohepatitis. Gastroenterology. 2008;135(4):1176-84.

17. Cui KQ, Zhao XW, Zhang Y, Kang XH, Meng J, Chen XL. Efficacy of rosiglitazone in treatment of nonalcoholic fatty liver disease and its relations with adiponectin. World chinese journal of digestology. 2006;14(13):1326 - 9.

18. Uygun A, Kadayifci A, Isik AT, Ozgurtas T, Deveci S, Tuzun A, et al. Metformin in the treatment of patients with non-alcoholic steatohepatitis. Alimentary pharmacology & therapeutics. 2004;19(5):537‐44.
